# Emergence of a Hybrid IncI1-Iα Plasmid-Encoded *bla*_CTX-M-101_ Conferring Resistance to Cephalosporins in *Salmonella enterica* Serovar Enteritidis

**DOI:** 10.3390/microorganisms11051275

**Published:** 2023-05-12

**Authors:** Xiaojie Qin, Zengfeng Zhang

**Affiliations:** 1 School of Health Science and Engineering, University of Shanghai for Science and Technology, Shanghai 200093, China; qxj19900709@163.com; 2 Department of Food Science & Technology, School of Agriculture and Biology, Shanghai Jiao Tong University, Shanghai 200240, China

**Keywords:** *Salmonella* Enteritidis, *bla*_CTX-M-101_, IncI1-Iα plasmid, IS*Ecp1*

## Abstract

The increasing resistance to cephalosporins in *Salmonella* poses a serious threat to public health. In our previous study, the *bla*_CTX-M-101_ gene, a new *bla*_CTX-M_ variant, was first reported in *Salmonella enterica* serovar Enteritidis (*S.* Enteritidis). Here, we further analyzed the genome characterization, transferability, and resistance mechanism of one *S.* Enteritidis isolate (SJTUF14523) carrying *bla*_CTX-M-101_ from an outpatient in 2016 in Xinjiang, China. This strain was a multidrug resistance (MDR) isolate and exhibited resistance to ceftazidime (MIC = 64 μg/mL), cefotaxime (MIC = 256 μg/mL), and cefepime (MIC = 16 μg/mL). Phylogenetic analysis revealed that SJTUF14523 had a close relationship to another *S.* Enteritidis isolate from the United States. In the presence of plasmid p14523A, there were 8- and 2133-fold increases in the MICs of cephalosporins in *Escherichia coli* C600 in the conjugation. Gene cloning results indicated that *bla*_CTX-M-101_ was the decisive mechanism leading to ceftazidime and cefotaxime resistance that could make the MICs break through the resistance breakpoint. Plasmid sequencing revealed that the *bla*_CTX-M-101_ gene was located on an IncI1-Iα transferable plasmid (p14523A) that was 85,862 bp in length. Sequence comparison showed that p14523A was a novel hybrid plasmid that might have resulted from the interaction between a homologous region. Furthermore, we found a composite transposon unit composed of IS*Ecp1*, *bla*_CTX-M-101_, and *orf*477 in p14523A. IS*Ecp1*-mediated transposition was likely to play a key role in the horizontal transfer of *bla*_CTX-M-101_ among plasmids in *S.* Enteritidis. Collectively, these findings underline further challenges in the prevention and control of antibiotic resistance posed by new CTX-M-101-like variants in *Salmonella*.

## 1. Introduction

The increased resistance to antibiotics in bacteria has become a global clinical and public health concern. It has been estimated that approximately 10 million people will die annually by 2050 due to drug-resistant bacteria unless there is a global response to the problem of antimicrobial resistance (AMR) [1]. Currently, cephalosporins are a common and effective first-line drug for infections by pathogens [2,3]. However, the cephalosporins resistance in *Enterobacteriaceae* has been frequently reported due to the abuse of this drug in clinical treatment and animal breeding [4,5]. The results from a previous study indicated that the extended-spectrum cephalosporin resistance increased from 5.46% to 12.97% between 2009 and 2016 in invasive *Escherichia coli* infections in hospitalized patients in the US [6]. Similar reports from China also showed that there was an increase in cefotaxime resistance from 52.2% in 2005 to 62.0% in 2014 in *E. coli* from inpatients and outpatients [7].

Notably, *Salmonella* is recognized as an important bacterial pathogen causing gastroenteritis worldwide, and it has been reported that this pathogen causes 115 million human infections and 370,000 deaths per year globally [8]. *Salmonella* has been classified into more than 2600 serovars based on their unique surface antigens [9]. Among them, *Salmonella enterica* serovar Enteritidis (*S.* Enteritidis) is one of the most common causative agents of salmonellosis [10,11,12]. Recently, serious antibiotic resistance—especially MDR in *S.* Enteritidis—has been continuously reported, posing a serious threat to food safety and public health. In particular, a relatively high incidence of resistance to cefotaxime (70.64%), cefepime (58.72%), and ceftazidime (48.62%) was reported in *S.* Enteritidis with an ACSSuT (i.e., ampicillin, chloramphenicol, streptomycin, sulfamethoxazole, and tetracycline) resistance pattern from patients [13]. Therefore, the dramatic increase in cephalosporin resistance requires the alteration of treatment strategies. It is vital to understand the mechanism of resistance to cephalosporins in *S.* Enteritidis for the prevention and control of pathogens.

The main mechanism of the resistance to cephalosporins is most often mediated by extended-spectrum β-lactamases (ESBLs) produced by pathogens, and CTX-M-β-lactamase is the most common type in *Salmonella*, *Escherichia coli*, and *Klebsiella pneumoniae* in China [14,15,16]. Among various *bla*_CTX-M_ subtypes, *bla*_CTX-M-14_ has always been the most common type both in *E. coli* and *K. pneumoniae* in China [16,17]. In our previous study, *bla*_CTX-M-55_ was identified to be the most common type in *S.* Enteritidis [14,18]. Notably, *bla*_CTX-M_ variants were always located on the transferable plasmid, which could potentially accelerate their dissemination among *Enterobacteriaceae* [14,18]. In the selective pressure of antibiotics, new variants of *bla*_CTX-M_ have been frequently reported in recent years, including *bla*_CTX-M-14.2_, *bla*_CTX-M-15.2_, *bla*_CTX-M-151_, *bla*_CTX-M-166_, *bla*_CTX-M-190_, *bla*_CTX-M-213_, and *bla*_CTX-M-236_ [19,20,21,22,23,24]. There are at least 170 unique CTX-M variants from plasmids or chromosomes recognized in *Enterobacteriaceae*, including in *Escherichia*, *Klebsiella*, and *Enterobacter* species [25].

Mobile genetic elements such as plasmids, insertion sequences, and integrons are generally considered to be the major driving force in the transmission of antibiotic-resistance genes in bacteria [26,27,28]. Previous studies indicated that plasmids with IncHI2, IncF, IncI1, IncP, IncN, and IncA/C replicons were often related to MDR *Salmonella* strains. Moreover, these plasmid types were found to be co-residents in several MDR *Salmonella* [27,28]. Conjugative plasmids facilitate the horizontal transfer of the *bla*_CTX-M_ gene to other isolates and even the crossing of species barriers [14,18]. More importantly, *bla*_CTX-M_ is usually located on conjugative plasmids that facilitate its transfer among *Enterobacteriaceae* [14,18]. The primary conjugative plasmid families in which *bla*_CTX-M-55_ has been found include IncF [29], IncI1 [30], IncN [31], and IncHI2 [32]. An important feature of the *bla*_CTX-M_ plasmids is the frequent existence of insertion sequences such as IS*Ecp1*, IS*26*, and IS*903* [33]. Among them, IS*Ecp1* is a heterogeneous class of mobile elements that can promote the translocation of antimicrobial resistance genes [34,35,36]. Whole-genome sequencing (WGS) has been widely applied to reveal the evolution, transmission, and mobile element characteristics of antibiotic-resistant bacteria. For example, the international spread of the MDR *Salmonella* Indiana ST17 isolates was revealed among several countries such as China, the United Kingdom, and the United States by the WGS technique [37]. Identification and genetic arrangement of fosfomycin and cephalosporin resistance determinants in clinical *S.* Enteritidis isolates were explored by complete plasmid sequencing [14].

A new CTX-M variant, namely the *bla*_CTX-M-101_ gene, was reported in one *S.* Enteritidis isolate from an outpatient in 2016 in Xinjiang, China, in our previous study [18]. However, the resistance mechanism and transfer characteristics of this variant have not been fully elucidated. Hence, in this study, we investigated the sequence characterization and horizontal transfer of *bla*_CTX-M-101_ through whole-genome sequencing and conjugation, and its contribution to antibiotic resistance was also explored.

## 2. Materials and Methods

### 2.1. Bacterial Strains

The *S.* Enteritidis isolate SJTUF14523 was recovered from the stool samples of an outpatient with diarrhea in Xinjiang, China, in 2016. This isolate was identified via API20E test strips (BioMerieux, Marcy-l’Étoile, France) and serotyped via commercial antiserum (Statens Serum Institute, Copenhagen, Denmark) according to the manufacturer’s guidelines. *Escherichia coli* (DH5α and ATCC 25922), *Enterococcus faecalis* ATCC 29212, and *Salmonella* Braenderup H9812 were also used in this study for subsequent experiments including antimicrobial susceptibility testing, gene function analysis, and S1-PFGE analysis. All strains were stored at −80 °C in Luria–Bertani (LB) broth containing 50% glycerol and propagated twice overnight before use.

### 2.2. Antimicrobial Susceptibility Testing

Antimicrobial susceptibility testing was performed on the SJTUF14523 isolate using the agar dilution method as recommended by the Clinical and Laboratory Standard Institute (CLSI; 2019). Briefly, a stock solution (5120 μg/mL) of antibiotics was prepared and diluted to different concentrations with specific solvents according to CLSI. Then, different concentrations of antibiotics were added to sterilized Mueller–Hinton (MHA) medium cooled to 45–50 °C to prepare antibiotic plates. The *S.* Enteritidis SJTUF14523 cells were inoculated on the LB agar overnight culture at 37 °C. The bacteria were wiped with a sterile cotton swab and diluted in sterile normal saline. In addition, the suspension of bacteria was adjusted to approximately 0.5 McFarland turbidity and inoculated onto MHA plates without antibiotics (as a control) and with different concentrations of antibiotics for cultivation for 18–24 h at 37 °C. Antibiotic susceptibility was interpreted by using MIC values based on CLSI. The following antibiotics were tested: cefoxitin (FOX), cefotaxime (CTX), ceftazidime (CAZ), cefepime (FEP), meropenem (MEM), nalidixic acid (NAL), trimethoprim–sulfamethoxazole (SXT), amikacin (AMK), ampicillin (AMP), gentamicin (GEN), ciprofloxacin (CIP), kanamycin (KAN), azithromycin (AZM), ofloxacin (OFX), tetracycline (TET), chloramphenicol (CHL), and streptomycin (STR). In addition, polymyxin B (PB) and polymyxin E (PE) testing were performed with broth microdilution as recommended by the European Committee on Antimicrobial Susceptibility Testing (EUCAST; 2019). All antibiotics used in this study were purchased from Sigma-Aldrich Shanghai Trading Co. Ltd., China. *E. coli* ATCC 25,922 and *E. faecalis* ATCC 29,212 were used as quality control isolates.

### 2.3. Whole-Genome Sequencing, Assembling, and Annotation

The *S.* Enteritidis SJTUF14523 cells were transferred to LB broth and incubated overnight at 37 °C and 200 rpm on a shaking incubator with a rotational radius of 26 mm. Genomic DNA was extracted from overnight cultures using the QIAamp DNA mini kit (Qiagen, CA). WGS was performed by the Personal Biotechnology Company (Shanghai, China) using a PacBio RS II system (Pacific Biosciences, Menlo Park, CA, USA) and the Illumina MiSeq (Illumina, San Diego, CA, USA). For the PacBio RS II platform, a 10 kbp DNA library was constructed and sequenced using single-molecule real-time (SMRT) sequencing technology. The sequence data of the PacBio RS II platform were assembled using Canu software (https://github.com/marbl/canu (accessed on 8 December 2022)) [38]. For the Illumina MiSeq platform, a 400 bp DNA library was constructed and sequenced in paired-end sequencing mode. The data from the Illumina MiSeq platform were assembled using SPAdes [39]. Finally, the consensus genome sequence was determined using Pilon software (https://github.com/broadinstitute/pilon (accessed on 8 December 2022)) [40].

Annotation of the genome was performed using the RAST [41], BLASTn, and BLASTp (http://blast.ncbi.nlm.nih.gov/Blast.cgi (accessed on 8 December 2022)) programs. The encoding genes in the genome were predicted by Glimmer [42] and GeneMarkS [43]. tRNAs, rRNAs, and repeated sequences in the genome were predicted using tRNAscan-SE v2.0 software (http://trna.ucsc.edu/software/ (accessed on 8 December 2022)), Barrnap (https://github.com/tseemann/barrnap (accessed on 8 December 2022)), and Tandem Repeats Finder v4.09 software (http://tandem.bu.edu/trf/trf.html (accessed on 8 December 2022)), respectively. The plasmid type was identified using Plasmidfinder [44].

### 2.4. Phylogenetic Analysis

The genome sequence in this study and genome sequences screened from *Salmonella* with cephalosporins resistance in the PATRIC database (https://patricbrc.org/ (accessed on 15 December 2022)) were used to establish phylogenetic trees. Single-nucleotide polymorphisms (SNPs) were extracted using Snippy (https://github.com/tseemann/snippy (accessed on 15 December 2022)) to generate the core genomic alignment. Gubbins was then used to identify and remove recombination regions using an algorithm that iteratively identified loci containing elevated densities of base substitutions, and then the resulting pairwise SNP differences were calculated [45]. The core SNP alignment was used to generate a maximum-likelihood phylogeny using RAxML v8.1.23 [46] with the GTR nucleotide substitution model. We also conducted 100 random bootstrap replicates to assess the node support. The display, annotation, and management of phylogenetic trees were performed using the ITOL tool [47].

### 2.5. Gene Cloning

Primers (F-GGAATTCATGGTTAAAAAATCACTGCG; R-CGAGCTCTCCGTTTCCGCTATTACA) with enzyme digestion sites of *Eco*RI and *Sac*I were designed using Primer Premier 5 software to amplify the *bla*_CTX-M-101_ fragment. Similarly, primers (F-GGAATTCTGAAAAGCGTGGTAATGC; R-CGAGCTCGTGGCTGCCGATGACTAT) were designed to amplify *bla*_CTX-M-101_ and its promoter region in the upstream of *bla*_CTX-M-101_ and named CTX-M-101P. Promoter sequences were predicted with the BPROM program (http://www.softberry.com/ (accessed on 8 February 2023)). The *bla*_CTX-M-101_ and CTX-M-101P fragments were amplified via PCR and cloned to the pMD19-T vector, yielding the recombinant plasmids pMD19-T-CTX-M-101 and pMD19-T-CTX-M-101P. These recombinant plasmids were transformed into *E.coli* DH5α competent cells using the electroporation method. Transformants were selected on MacConkey agar plates supplemented with 4 µg/mL cefotaxime and 50 µg/mL ampicillin.

### 2.6. Plasmid Conjugation and Transformation Experiment

Conjugation experiments were performed as previously described with *E. coli* C600 as the recipient [32]. Briefly, *Salmonella* used as the donor was incubated with the recipient overnight, mixed, and transferred to the filter membrane on an LB plate for overnight culture. Transconjugants were selected on MacConkey agar plates supplemented with cefotaxime (4 µg/mL) and rifampin (200 µg/mL). Transconjugants were further identified using 16S RNA and PCR.

For the transformation experiments, plasmid DNA from the XDR isolates was extracted using the Qiagen Plasmid Midi Kit according to the manufacturer’s instructions (Qiagen GmbH, Hilden, Germany). The purified plasmid was transformed into *E. coli* DH5α (Takara Biotechnology, Dalian, China) cells. The transformants were selected using MacConkey agar with cefotaxime (4 µg/mL). The MICs of the transconjugants and transformants were tested using the agar dilution method as recommended by CLSI 2019.

### 2.7. S1-PFGE Experiment

Pulsed-field gel electrophoresis (PFGE) with S1 nuclease (Takara Biotechnology, Dalian, China) digestion was carried out to determine the size of the plasmid. Briefly, after cells (OD600 = 0.95 − 1.00) were fixed with SeaKem Gold agarose (Cambrex BioScience, Walkersville, MD, USA) and subsequently lysed, the embedded DNAs were digested using 18 U S1 enzymes (Takara Biotechnology, Dalian, China) in a 37 °C water bath for 15 min. The restricted DNA fragments were separated in 0.5 × TAE buffer at 14 °C for 19 h using a CHEF Mapper electrophoresis system (Bio-Rad, Richmond, CA, USA) with pulse times of 2.16–63.8 s. The PFGE image was obtained with a Gel Imager System (Bio-Rad, USA). *S.* Braenderup H9812 was used as the DNA size marker.

### 2.8. Nucleotide Sequence Accession Numbers

The complete sequences of the *S.* Enteritidis SJTUF14523 chromosome (accession number CP074428), p14523A (accession number CP074429), p14523B (accession number CP074430), and p14523C (accession number CP074431) were deposited in the NCBI database.

## 3. Results and Discussion

### 3.1. Emergence of bla_CTX-M-101_ in S. *Enteritidis* Isolate

In the investigation of the ESBL CTX-M subtype in *Salmonella* in China, we identified that *S.* Enteritidis SJTUF14523 carried *bla*_CTX-M-101_, a new CTX-M subtype. Antimicrobial susceptibility testing showed that the SJTUF14523 isolate exhibited resistance to ceftazidime (MIC = 64 μg/mL), cefotaxime (MIC = 256 μg/mL), and cefepime (MIC = 16 μg/mL) (Table 1), and this isolate was also resistant to ampicillin (MIC ≥ 128 μg/mL), nalidixic acid (MIC ≥ 128 μg/mL), trimethoprim–sulfamethoxazole (MIC ≥ 16/304 μg/mL), and kanamycin (MIC ≥ 128 μg/mL). Therefore, SJTUF14523 was an MDR isolate. Whole-genome sequencing was then performed on this isolate. Antibiotic resistance genes and chromosome point mutation were consistent with the presented resistance to β-lactams (*bla*_CTX-M-101_ and *bla*_TEM-1b_), aminoglycosides (*aac(6′)-Iaa*, *aph(3″)-Ib*, and *aph(6)-Id*), sulfonamides (*sul2*), and quinolones (*gyr*AD87Y) (Table S1).

Extended-spectrum cephalosporins are the primary drugs of choice for treating salmonellosis; however, there is a rising emergence of *Salmonella* resistance to these antibiotics due to their abuse and overuse in humans and livestock [48,49,50,51]. In 2013, the total amount of antibiotics used in China was about 162,000 tons, approximately 160 times that of the United Kingdom, and 48% of which was used for human consumption; the rest was shared by animals [52]. Furthermore, the production yields of fluoroquinolones (including ciprofloxacin) and β-lactams (including ceftriaxone) in China were estimated to be 27,300 and 34,100 tons, respectively [52]. Therefore, governmental regulations limiting the use of antimicrobial agents have been issued in China to reduce the potential threat of MDR bacteria to public health.

In this study, *S.* Enteritidis SJTUF14523 exhibited resistance to ceftazidime, cefotaxime, cefepime, ampicillin, nalidixic acid, trimethoprim–sulfamethoxazole, and kanamycin, indicating its MDR. Moreover, this isolate carried some antibiotic resistance genes that included the novel CTX-M type gene *bla*_CTX-M-101_. Exploring the function of this gene is important for understanding the transmission mechanism of MDR *Salmonella*.

### 3.2. bla_CTX-M-101_ Mediated the Resistance to Cephalosporin

The effect of *bla*_CTX-M-101_ on cephalosporin resistance was further explored through conjugation, transformation, and gene cloning. Plasmid p14523A was successfully transferred into *E. coli* C600 as the recipient through conjugation. There were 8- and 2133-fold increases in the MICs of cephalosporins, including ceftazidime (256-fold increase in MIC), cefotaxime (approximately 2133-fold increase in MIC), cefepime (approximately 267-fold increase in MIC), and cefoxitin (8-fold increase in MIC) in *E. coli* C600 with the presence of p14523A. Similar results were also observed in *E. coli* DH5α after obtaining the transformed p14523A. We then constructed two recombinant plasmids of pMD19-T-CTX-M-101 and pMD19-T-CTX-M-101P. Based on pMD19-T-CTX-M-101, we added the promoter region of *bla*_CTX-M-101_, yielding pMD19-T-CTX-M-101P. The presence of pMD19-T-CTX-M-101 resulted in 2- and 2133-fold increases in the MICs of cephalosporins, including ceftazidime (128-fold increase in MIC), cefotaxime (approximately 2133-fold increase in MIC), cefepime (approximately 133-fold increase in MIC), and cefoxitin (2-fold increase in MIC). pMD19-T-CTX-M-101P resulted in 2- and 16-fold higher increases in cephalosporin MICs than pMD19-T-CTX-M-101, suggesting that its protomers facilitated the expression of *bla*_CTX-M-101_.

Cephalosporin resistance is usually due to the ESBLs produced by *Enterobacteriaceae* [50]. The *bla*_CTX-M_ gene is generally located on transferable plasmids that could facilitate the dissemination among *E.coli*, *Salmonella*, and other pathogens [50,53,54,55]. The *bla*_CTX-M-14_ subtype has been found in *Salmonella* Indiana isolates from chickens and pigs in Guangdong [56] and also in *Salmonella* Typhimurium isolates from humans [50]. The *bla*_CTX-M-65_ subtype was the predominant type in *Salmonella* Indiana isolates from humans and food-producing animals in Henan [48]. To the best of our knowledge, this is the first report that *bla*_CTX-M-101_ was found in *S.* Enteritidis isolates, which sounds an alarm regarding the control of the emergence of new antimicrobial resistance gene variants in bacteria.

### 3.3. Phylogenetic Analysis of bla_CTX-M-101_-Positive S. Enteritidis Isolate

**Figure 1:**
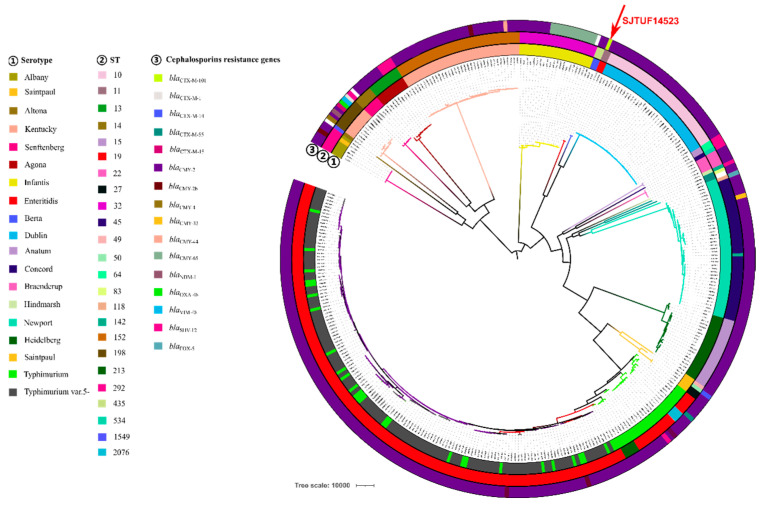
Maximum likelihood phylogenetic tree of 404 *Salmonella* isolates. There are three rings noted as ①–③ from the inner to the outer in the tree. The detailed information in different rings is colored (see the key). The SITUF14523 isolate in this study is identified by the red arrow.

A total of 11,629 core SNPs were identified in 404 genomes of *Salmonella* isolates with resistance to cephalosporins, then these core SNPs were used to build an ML phylogenetic tree as shown in Figure 1. A total of 18 serotypes and 24 sequence types (STs) were identified in these 404 genomes. These 18 serotypes were Enteritidis, Albany, Saintpaul, Altona, Kentucky, Senftenberg, Agona, Infantis, Berta, Dublin, Anatum, Concord, Braenderup, Hindmarsh, Newport, Heidelberg, Typhimurium, and Typhimurium var.5-. These 24 STs were ST10, ST11, ST13, ST14, ST15, ST19, ST22, ST27, ST32, ST45, ST49, ST50, ST64, ST83, ST118, ST142, ST152, ST198, ST213, ST292, ST435, ST534, ST1549, and ST2076. The clones of *Salmonella* isolates in the phylogenetic tree were consistent with the serotypes (Figure 1). Various *bla*_CTX-M_ and *bla*_CMY_ subtypes were identified in these genomes, but *bla*_CTX-M-101_ was only identified in SJTUF14523, suggesting its specificity. It was interesting that SJTUF14523 in this study was clustered with another *S.* Enteritidis 2014AM-1411 from the United States. Both SJTUF14523 and 2014AM-1411 belonged to ST11 and exhibited resistance to third-generation cephalosporins. SJTUF14523 was identified to carry *bla*_CTX-M-101_, but 2014AM-1411 carried *bla*_CMY-2_. The branch of these two *S.* Enteritidis isolates was adjacent to those of *Salmonella* Berta and *Salmonella* Dublin, suggesting their close genetic relationship.

In addition, the *Salmonella* Infantis isolates shown in Figure 1 belonged to ST32, and this clone carried the cephalosporin resistance genes of *bla*_CMY-2_ and *bla*_CMY-65_. *Salmonella* Dublin belonged to ST10, and this clone carried *bla*_CMY-2_. *Salmonella* Newport mainly belonged to ST45, and this clone mainly carried *bla*_CMY-2_. *Salmonella* Heidelberg belonged to ST15, and this clone mainly carried *bla*_CMY-2_. *Salmonella* Typhimurium was clustered with Typhimurium var.5- (Figure 1), both of which mainly belonged to ST19, and this clone mainly carried *bla*_CMY-2_. A part of *Salmonella* Typhimurium belonged to ST2076, and some belonged to ST213, both of which were uncommon STs. *Salmonella* Kentucky isolates were divided into two clades: one clade belonged to ST198, and another clade belonged to ST152. A variety of ESBL genes including *bla*_CTX-M-15_, *bla*_CTX-M-1_, *bla*_CMY-4_, *bla*_CMY-2_, *bla*_CMY-16_, *bla*_OXA-48_, and *bla*_VIM-48_ were identified in the ST198 clone. The ST152 clone mainly carried *bla*_CMY-2_.

### 3.4. Characterization of a Novel Hybrid Plasmid Carrying bla_CTX-M-101_

**Figure 2:**
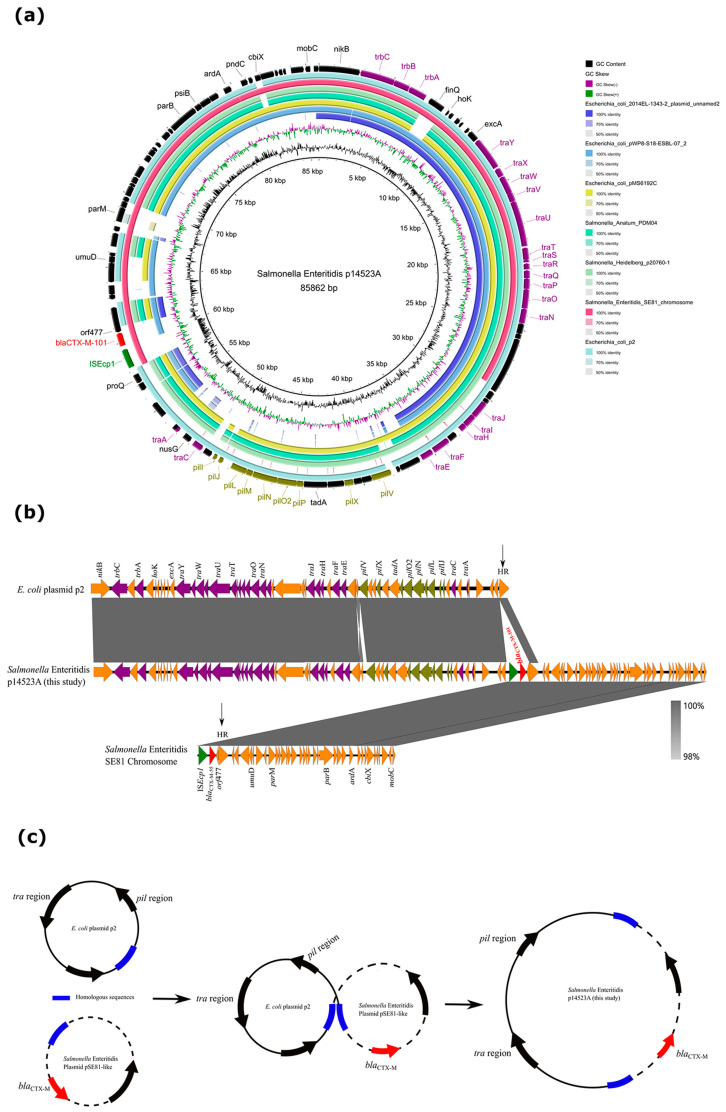
(**a**) Sequence comparison of plasmid p14523A and other plasmids including *Escherichia coli* 2014EL-1343-2 plasmid unnamed2 (accession number NZ_CP024230), pWP8-S18-ESBL-07_2 (accession number NZ_AP022263), pMS6192C (accession number NZ_CP054943), p2 (accession number CP028485), Salmonella Anatum PDM04 (accession number NZ_CP013224), and *Salmonella* Heidelberg p20760-1 (accession number NZ_CP051411). (**b**) Linearized comparison of plasmid p14523A, *E. coli* p2 (Accession no CP028485), and *S.* Enteritidis SE81 Chromosome (accession number NZ_CP050721) using Easyfig. (**c**) Potential mechanism of plasmid fusion through homologous recombination. Blue, homologous region; red, antibiotic resistance gene; black, others.

The characterization of plasmids was further analyzed in SJTUF14523. There were three plasmids in SJTUF14523: p14523A, p14523B, and p14523C (Table S1). Furthermore, *bla*_CTX-M-101_ was located on plasmid p14523A, which possessed an IncI1-Iα plasmid structure with GC content of 50.0% and was 85,862 bp in length. The Blastn results showed that p14523A was similar to *Escherichia coli* pMS6192C (89% coverage; 98.87% identity), p2 (91% coverage; 98.93% identity), *Salmonella* Anatum PDM04 (86% coverage; 97.82% identity), and *Salmonella* Heidelberg p20760-1 (87% coverage; 97.81% identity). All of these plasmids harbored the IncI1-Iα replication gene and possessed similar conjugation, maintenance, and stability function regions (Figure 2a). However, variable regions containing *parM* and *umuD* genes and an insert sequence (IS*Ecp1*) as well as an antibiotic resistance gene (*bla*_CTX-M-101_) were special to p14523A. It was interesting that these variable regions showed a high similarity to those of the chromosome sequence from *S.* Enteritidis SE81 but differed by *bla*_CTX-M-55_ in SE81 (Figure 2a). The SE81 chromosome sequence showed a high similarity to a plasmid structure containing conjugation, maintenance, and stability function regions, suggesting that it might have originated from plasmids.

Further analysis showed that p14523A was a hybrid plasmid through a chimera process of the p2 and SE81 chromosomes. The p2 and SE81 sequences might have formed a hybrid plasmid through interaction between a homologous region (HR), namely a 1394 bp DNA sequence encoding a hypothetical protein (Figure 2b). We proposed that two daughter plasmids (p2 and pSE81-like plasmid) were aligned at the HR sequence, then homologous recombination activities occurred and finally formed a hybrid plasmid (Figure 2c). Currently, plasmid fusion often occurs during bacterial conjugation, and this is often mediated by insertion sequences (IS*26*) [57,58]. However, p14523A was not formed in the conjugation, and it pre-existed in the original isolate. Therefore, the hybrid plasmid in this study was different from those in the previous studies, and it is urgent to control and prevent the dissemination of p14523A-like plasmids among *Enterobacteriaceae*.

### 3.5. Composite-Transposon-Mediated Capture of bla_CTX-M-101_ by Plasmid

**Figure 3:**
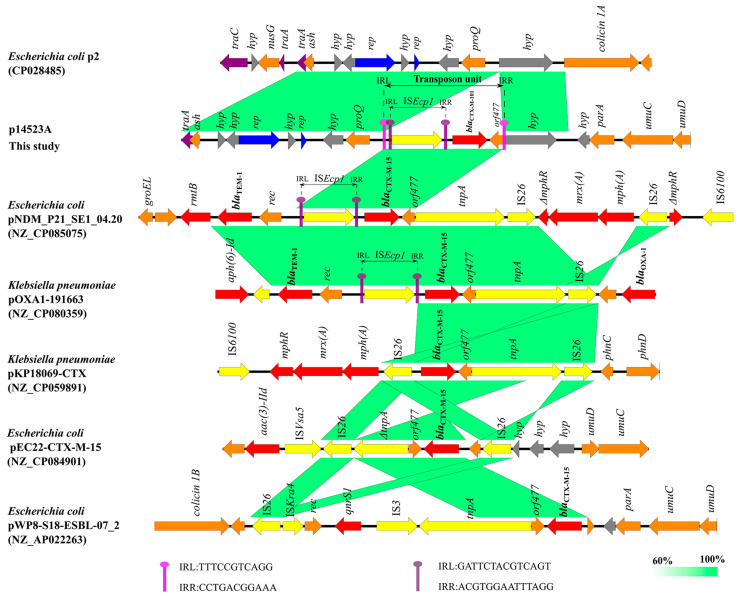
Genetic environment of *bla*_CTX-M-101_ in plasmid p14523A (this study), *E. coli* p2, pNDM_P21_SE1_04.20, pEC22-CTX-M-15, pWP8-S18-ESBL-07_2, *K. pneumoniae* pOXA1-191663, and pKP18069-CTX. Areas shaded in green indicate homologies between the corresponding genetic loci on each plasmid. Boxes or arrows represent the ORFs. Red, antibiotic resistance genes; yellow, IS/transposase; gray, hypothetical protein; blue, replicon; brown, other genes.

To explore the horizontal transfer of *bla*_CTX-M-101_, we analyzed its genetic environment and compared its sequence with that of *bla*_CTX-M-15_ (Figure 3). We found that the difference between *bla*_CTX-M-101_ and *bla*_CTX-M-15_ was one base at position 377 (T377G), yielding an amino acid mutation at position 126 (I126S) (Figure S1). The genetic environments of *bla*_CTX-M-101_ and *bla*_CTX-M-15_ were similar in p14523A, *E. coli* pNDM_P21_SE1_04.20, and *K. pneumoniae* pOXA1-191663. The upstream of both *bla*_CTX-M-101_ and *bla*_CTX-M-15_ was IS*Ecp1*, and the downstream was *orf*477 in these plasmids. However, *bla*_CTX-M-15_ could also form a longer, more flexible, and complex transposon structure together with other antibiotic resistance genes such as *rmtB*, *mph(A)*, *bla*_TEM_, *aac(3′)-Iid*, and *qnrS1* with the help of IS*26*, IS*6100*, and *tnpA* (Figure 3). Compared with *E. coli* p2, *bla*_CTX-M-101_ was apparently captured by p12523A. The *bla*_CTX-M-101_ gene, IS*Ecp1*, and *orf*477 formed a composite transposon unit in p14523A. IRL (TTTCCGTCAGG) and IRR (CCTGACGGAAA) were found at the end of this transposon unit, providing evidence for traces of transposon. We then proposed that the process of co-integrating into a plasmid was much more likely mediated by the transposon IS*Ecp1*. IS*Ecp1* appeared to be able to use IRL in combination with a sequence beyond its IRR end to move an adjacent region, yielding the transfer of the entire transposon unit [59,60]. IS*Ecp1*-mediated transposition of *bla*_CTX-M-64_, *bla*_CTX-M-2_, *bla*_CTX-M-3_, and *bla*_TEM-1b_ have been demonstrated [61,62,63]. Moreover, IS*Ecp1* could capture DNA regions with different sizes and simultaneously transfer adjacent regions [59]. Therefore, IS*Ecp1*-mediated transposition could be responsible for capturing *bla*_CTX-M-101_ by the IncI1-Iα plasmid.

## 4. Conclusions

In summary, our study highlighted the emergence of *bla*_CTX-M-101_, a new *bla*_CTX-M_ variant, in *S.* Enteritidis. The *bla*_CTX-M-101_ gene mediated the resistance to third-generation cephalosporin (ceftazidime and cefotaxime). The *bla*_CTX-M-101_ gene was located on an IncI1-Iα transferable plasmid p14523A that facilitated its spread among *Enterobacteriaceae* through bacterial conjugation. This IncI1-Iα plasmid appeared to be very active and could fuse DNA fragments from other plasmids or chromosomes by activating homologous recombination. We also identified the transposition event driven by IS*Ecp1* in this plasmid, which was likely to be responsible for the capture and transfer of *bla*_CTX-M-101_ among different plasmids in *Enterobacteriaceae*. The possibility of dissemination of these CTX-M-101-like variants and their transferable plasmids among *Enterobacteriaceae* should be an important consideration in the “One Health” system.

## Figures and Tables

**Table 1:** MICs of cephalosporins in the parental strain, transconjugant (C600 strains), and transformants(DH5α strains).

Strains	MIC				Plasmid Types	Plasmid Sizes (Kb)
	FOX	CAZ	CTX	FEP		
***S.* Enteritidis strain**						
SJTUF14253	8	64	256	16	I1-Iα, FIIs-FIB, X1	~22, ~65, ~85
***E. coli* strains**						
C600	2	0.125	0.06	0.03		
C600/p14523A (CTX-M-101)	16	32	128	8	I1-Iα	~85
DH5α	2	0.25	0.03	0.03		
DH5α/p14523A (CTX-M-101)	8	32	128	8	I1-Iα	~85
DH5α/pMD19-T	2	0.25	0.03	0.03		
DH5α/pMD19-T-CTX-M-101	4	32	64	4		
DH5α/pMD19-T-CTX-M-101P ^a^	8	64	256	64		

^a^ CTX-M-101P stands for the nucleotide region containing the CTX-M-101 sequence and its promoter sequence. FOX, cefoxitin; CAZ, ceftazidime; CTX, cefotaxime; FEP, cefepime.

## Data Availability

The data of this study are available from the authors upon reasonable request.

## Supplementary Materials

The following supporting information can be downloaded at: https://www.mdpi.com/article/10.3390/microorganisms11051275/s1, Figure S1: Nucleotide sequence (a) and amino acid sequence (b) comparison of *bla*_CTX-M-101_ and *bla*_CTX-M-15_. Figure S2: Plasmid profiles of *S.* Enteritidis SJTUF14523 and its transconjugant (SJTUF14523-TC) determined via S1-PFGE. Table S1: Resistance genes and plasmids carried by *S.* Enteritidis SJTUF14253.
