# Supplementary material for: Emergence of a Hybrid IncI1-Iα Plasmid-Encoded blaCTX-M-101 Conferring Resistance to Cephalosporins in Salmonella enterica Serovar Enteritidis
Source: Microorganisms. 2023 May 12;11(5):1275. doi: 10.3390/microorganisms11051275 (PMC10222410; doi:10.3390/microorganisms11051275)
Supplement: Supplementary file 1 [file microorganisms-11-01275-s001.zip › Supplementary Figure S1.pdf]

(a)

Nucleotide sequence

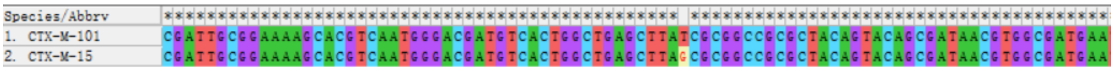

(b)

Amino acid sequence

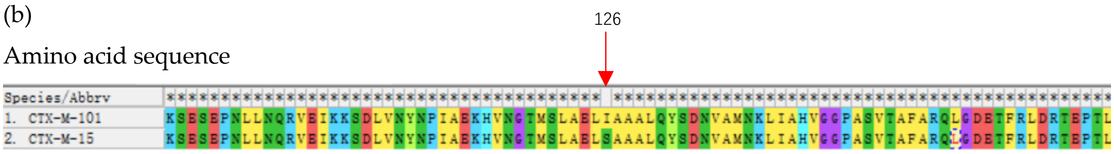

Figure S1 Nucleotide sequence (a) and amino acid sequence (b) comparison of *bla*$_{CTX\text{-}M\text{-}101}$ and *bla*$_{CTX\text{-}M\text{-}15}$
