# Supplementary material for: Emergence of a Hybrid IncI1-Iα Plasmid-Encoded blaCTX-M-101 Conferring Resistance to Cephalosporins in Salmonella enterica Serovar Enteritidis
Source: Microorganisms. 2023 May 12;11(5):1275. doi: 10.3390/microorganisms11051275 (PMC10222410; doi:10.3390/microorganisms11051275)
Supplement: Supplementary file 1 [file microorganisms-11-01275-s001.zip › Supplementary Figure S2.pdf]

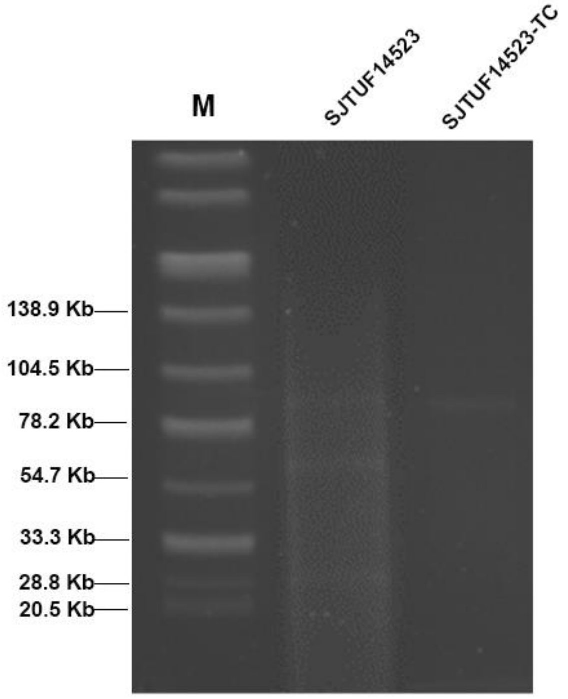

Figure S2 Plasmid profiles of *S*. Enteritidis SJTUF14523 and its transconjugant (SJTUF14523-TC) determined by S1-PFGE. Lanes M, H9812 was used as molecular size marker with different bands labeled
