# Supplementary material for: Emergence of a Hybrid IncI1-Iα Plasmid-Encoded blaCTX-M-101 Conferring Resistance to Cephalosporins in Salmonella enterica Serovar Enteritidis
Source: Microorganisms. 2023 May 12;11(5):1275. doi: 10.3390/microorganisms11051275 (PMC10222410; doi:10.3390/microorganisms11051275)
Supplement: Supplementary file 1 [file microorganisms-11-01275-s001.zip › Supplementary Table S1.pdf]

Table S1 Resistance genes and plasmids carried by *S*. Enteritidis SJTUF14253

	**Chromosome**	**Plasmids**		
		**p14253A**	**p14253B**	**p14253 C**
Size (bp)	4,679,991	85,862	64,327	24,486
Replicon type		IncI1	IncFII-FIB	IncX1
Resistance genes				
Aminoglycosides	*aac(6′)-Iaa*			*aph(3′′)-Ib*, *aph(6)-Id*
β-lactams		*bla*$_{CTX-M-101}$	*bla*$_{TEM-1b}$	*bla*$_{TEM-1b}$
Colistin				
Quinolones				
Fosfomycin				
Sulphonamides				*sul2*
Trimethoprim				
Phenicol				
Rifampicin				
Tetracyclines				
Chromosomal mutation	*gyr*AD87Y			
